# 2-Amino-5-methyl­pyridinium nicotinate

**DOI:** 10.1107/S1600536810005970

**Published:** 2010-02-20

**Authors:** Madhukar Hemamalini, Hoong-Kun Fun

**Affiliations:** a X-ray Crystallography Unit, School of Physics, Universiti Sains Malaysia, 11800 USM, Penang, Malaysia

## Abstract

In the title compound, C_6_H_9_N_2_^+^·C_6_H_4_NO_2_^−^, the 2-amino-5-methyl­pyridinium cation is essentially planar, with a maximum deviation of 0.023 (2) Å. In the crystal, the cations and anions are linked *via* strong N—H⋯O hydrogen bonds, forming a two dimensional network parallel to (100). In addition, π⋯π inter­actions involving the pyridinium and pyridine rings, with centroid–centroid distances of 3.6383 (8) Å, are observed.

## Related literature

For background to the chemistry of substituted pyridines, see: Pozharski *et al.* (1997 ▶); Katritzky *et al.* (1996 ▶). For nicotinic acid, see: Athimoolam & Rajaram (2005 ▶); Lorenzen *et al.* (2001 ▶); Gielen *et al.* (1992 ▶); Kim *et al.* (2004 ▶). For a related structure, see: Nahringbauer & Kvick (1977 ▶). For details of hydrogen bonding, see: Jeffrey & Saenger (1991 ▶); Jeffrey (1997 ▶); Scheiner (1997 ▶). For hydrogen-bond motifs, see: Bernstein *et al.* (1995 ▶). For bond-length data, see: Allen *et al.* (1987 ▶).
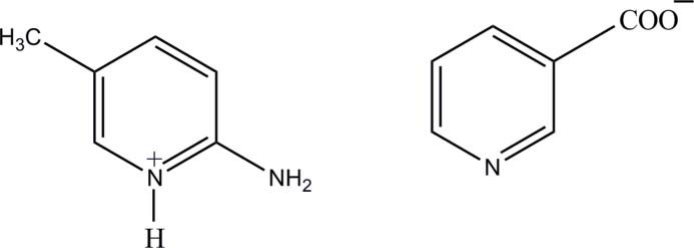


## Experimental

### 

#### Crystal data

C_6_H_9_N_2_^+^·C_6_H_4_NO_2_^−^
*M* *_r_* = 231.25
Monoclinic, 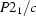

*a* = 9.4877 (3) Å
*b* = 11.1403 (3) Å
*c* = 11.7611 (3) Å
β = 110.113 (2)°
*V* = 1167.29 (6) Å^3^
*Z* = 4
Mo *K*α radiation
μ = 0.09 mm^−1^
*T* = 296 K
0.63 × 0.11 × 0.11 mm

#### Data collection

Bruker SMART APEXII CCD area-detector diffractometer
Absorption correction: multi-scan (*SADABS*; Bruker, 2009 ▶) *T*_min_ = 0.944, *T*_max_ = 0.990
14482 measured reflections
3870 independent reflections
2240 reflections with *I* > 2σ(*I*)
*R*_int_ = 0.026

#### Refinement

*R*[*F* ^2^ > 2σ(*F* ^2^)] = 0.050
*wR*(*F* ^2^) = 0.144
*S* = 1.05
3870 reflections
195 parameters
H atoms treated by a mixture of independent and constrained refinement
Δρ_max_ = 0.20 e Å^−3^
Δρ_min_ = −0.20 e Å^−3^

### 

Data collection: *APEX2* (Bruker, 2009 ▶); cell refinement: *SAINT* (Bruker, 2009 ▶); data reduction: *SAINT*; program(s) used to solve structure: *SHELXTL* (Sheldrick, 2008 ▶); program(s) used to refine structure: *SHELXTL*; molecular graphics: *SHELXTL*; software used to prepare material for publication: *SHELXTL* and *PLATON* (Spek, 2009 ▶).

## Supplementary Material

Crystal structure: contains datablocks global, I. DOI: 10.1107/S1600536810005970/sj2728sup1.cif

Structure factors: contains datablocks I. DOI: 10.1107/S1600536810005970/sj2728Isup2.hkl

Additional supplementary materials:  crystallographic information; 3D view; checkCIF report

## Figures and Tables

**Table 1:** Hydrogen-bond geometry (Å, °)

*D*—H⋯*A*	*D*—H	H⋯*A*	*D*⋯*A*	*D*—H⋯*A*
N1—H1*N*1⋯O2^i^	0.988 (16)	1.703 (16)	2.6899 (15)	176.8 (16)
N2—H1*N*2⋯O2^ii^	0.883 (16)	1.999 (16)	2.8756 (17)	171.7 (15)
N2—H2*N*2⋯O1^i^	0.936 (18)	1.878 (18)	2.8122 (17)	176.6 (17)

Symmetry codes: (i) 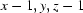; (ii) 
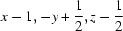
.

**Fig. 1.**
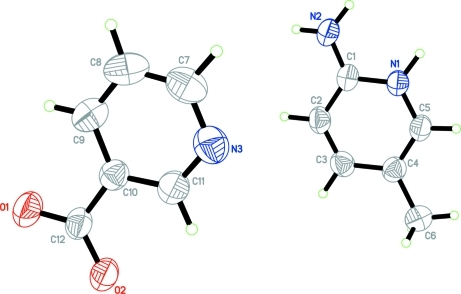
The asymmetric unit of the title compound. Displacement ellipsoids are drawn at the 50% probability level.

**Fig. 2.**
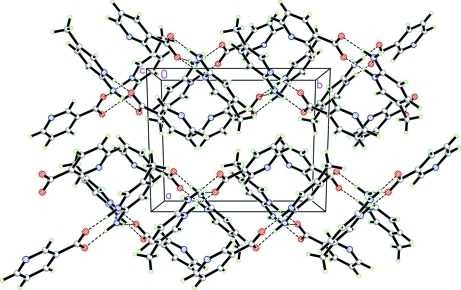
The crystal packing of the title compound, showing hydrogen-bonded (dashed lines) networks.

## supplementary crystallographic information

### Comment

Pyridine and its derivatives play important roles in heterocyclic chemistry (Pozharski *et al.*, 1997; Katritzky *et al.*, 1996). They are often  involved in hydrogen-bond interactions (Jeffrey & Saenger, 1991; Jeffrey, 1997; Scheiner, 1997). Nicotinic acid (vitamin B3), known as niacin, is a lipid lowering agent widely used to treat hypertriglyceridemia by the inhibition of lipolysis in adipose tissue (Athimoolam & Rajaram, 2005). The nicotinic acid complex 5-methylpyrazine-2-carboxylic acid-4-oxide is a commonly used drug for the treatment of hypercholesterolemia (Lorenzen *et al.*, 2001). Coordination complexes of nicotinic acid with metals such as Sn possess antitumour activity greater than the well known cisplatin or doxorubicin (Gielen *et al.*, 1992). The enzyme nicotinic acid mononucleotide adenyltransferase is essential for the synthesis of nicotinamide adenine dinucleotide in all living cells and is a potential target for antibiotics (Kim *et al.*, 2004). Since our aim is to study some interesting hydrogen bonding interactions, the crystal structure of the title compound is presented here.

The asymmetric unit (Fig. 1) contains one 2-amino-5-methylpyridinium cation and one nicotinate anion. The proton transfer from the carboxyl group to atom N1 of 2-amino-5-methylpyridine resulted in the widening of C1—N1—C5 angle of the pyridinium ring to 122.61 (11)°, compared to the corresponding angle of 117.4° in neutral 2-amino-5-methylpyridine (Nahringbauer & Kvick, 1977). The 2-amino-5-methylpyridinium cation is essentially planar, with a maximum deviation of 0.023 (2)Å for atom C6. The bond lengths are normal (Allen *et al.*, 1987).

In the crystal packing (Fig. 2), the protonated N1 atom and the 2-amino group (N2) is hydrogen-bonded to the carboxylate oxygen atoms (O1 and O2) via a pair of intermolecular N1—H1N1···O2 and N2—H2N2···O1 hydrogen bonds forming an *R*_2_^2^(8) ring motif (Bernstein *et al.*, 1995). The intermolecular N2—H1N2···O2 hydrogen bonds connect these molecules into 2-dimensional networks parallel to the (100)-plane (see Table 1). The crystal structure is further stabilized by π···π interactions involving the pyridine (C7–C11/N3) and pyridinium (C1–C5/N1) rings, with centroid to centroid distance of 3.6383 (8)Å [symmetry code: 1-x, 1-y, 1-z].

### Experimental

A hot methanol solution (20 ml) of 2-amino-5-methylpyridine (54 mg, Aldrich) and nicotinic acid (62 mg, Merck) were mixed and warmed over a heating magnetic stirrer for a few minutes. The resulting solution was allowed to cool slowly at room temperature and crystals of the title compound appeared after a few days.

### Refinement

The methyl H atoms were positioned geometrically and were refined using a riding model, with *U*_iso_(H) = 1.5*U*_eq_(C). A rotating group model was used for the methyl group. The remaining H atoms were located in a difference map and refined freely [N–H = 0.883 (16)–0.988 (16)Å, C–H = 0.946 (13)–1.015 (17)Å].

### Crystal data

**Table:**

C_6_H_9_N_2_^+^·C_6_H_4_NO_2_^−^	*F*(000) = 488
*M**_r_* = 231.25	*D*_x_ = 1.316 Mg m^−^^3^
Monoclinic, *P*2_1_/*c*	Mo *K*α radiation, λ = 0.71073 Å
Hall symbol: -P 2ybc	Cell parameters from 4062 reflections
*a* = 9.4877 (3) Å	θ = 2.6–26.7°
*b* = 11.1403 (3) Å	µ = 0.09 mm^−^^1^
*c* = 11.7611 (3) Å	*T* = 296 K
β = 110.113 (2)°	Needle, colourless
*V* = 1167.29 (6) Å^3^	0.63 × 0.11 × 0.11 mm
*Z* = 4	

### Data collection

**Table:**

Bruker SMART APEXII CCD area-detector diffractometer	3870 independent reflections
Radiation source: fine-focus sealed tube	2240 reflections with *I* > 2σ(*I*)
graphite	*R*_int_ = 0.026
φ and ω scans	θ_max_ = 31.6°, θ_min_ = 2.3°
Absorption correction: multi-scan (*SADABS*; Bruker, 2009)	*h* = −13→13
*T*_min_ = 0.944, *T*_max_ = 0.990	*k* = −15→16
14482 measured reflections	*l* = −17→17

### Refinement

**Table:**

Refinement on *F*^2^	Primary atom site location: structure-invariant direct methods
Least-squares matrix: full	Secondary atom site location: difference Fourier map
*R*[*F*^2^ > 2σ(*F*^2^)] = 0.050	Hydrogen site location: inferred from neighbouring sites
*wR*(*F*^2^) = 0.144	H atoms treated by a mixture of independent and constrained refinement
*S* = 1.05	*w* = 1/[σ^2^(*F*_o_^2^) + (0.0668*P*)^2^ + 0.0299*P*] where *P* = (*F*_o_^2^ + 2*F*_c_^2^)/3
3870 reflections	(Δ/σ)_max_ < 0.001
195 parameters	Δρ_max_ = 0.20 e Å^−^^3^
0 restraints	Δρ_min_ = −0.20 e Å^−^^3^

### Special details

**Table:**

Geometry. All s.u.'s (except the s.u. in the dihedral angle between two l.s. planes) are estimated using the full covariance matrix. The cell s.u.'s are taken into account individually in the estimation of s.u.'s in distances, angles and torsion angles; correlations between s.u.'s in cell parameters are only used when they are defined by crystal symmetry. An approximate (isotropic) treatment of cell s.u.'s is used for estimating s.u.'s involving l.s. planes.
Refinement. Refinement of F^2^ against ALL reflections. The weighted R-factor wR and goodness of fit S are based on F^2^, conventional R-factors R are based on F, with F set to zero for negative F^2^. The threshold expression of F^2^ > 2σ(F^2^) is used only for calculating R-factors(gt) etc. and is not relevant to the choice of reflections for refinement. R-factors based on F^2^ are statistically about twice as large as those based on F, and R- factors based on ALL data will be even larger.

### Fractional atomic coordinates and isotropic or equivalent isotropic displacement parameters (Å^2^)

**Table:**

	*x*	*y*	*z*	*U*_iso_*/*U*_eq_	
N1	0.02188 (12)	0.19316 (10)	0.05859 (9)	0.0442 (3)	
N2	−0.10328 (14)	0.22434 (12)	0.19337 (12)	0.0585 (3)	
C1	−0.00012 (14)	0.16474 (11)	0.16305 (10)	0.0442 (3)	
C2	0.08966 (14)	0.07300 (12)	0.23423 (12)	0.0491 (3)	
C3	0.19225 (14)	0.01646 (12)	0.19651 (12)	0.0503 (3)	
C4	0.21339 (13)	0.04715 (12)	0.08710 (11)	0.0478 (3)	
C5	0.12592 (14)	0.13727 (12)	0.02171 (11)	0.0459 (3)	
C6	0.33142 (17)	−0.01226 (17)	0.04856 (14)	0.0688 (4)	
H6A	0.3313	0.0225	−0.0262	0.103*	
H6B	0.3105	−0.0966	0.0373	0.103*	
H6C	0.4280	−0.0007	0.1098	0.103*	
O1	0.72673 (12)	0.39748 (10)	1.02919 (8)	0.0659 (3)	
O2	0.86277 (12)	0.36701 (9)	0.91118 (8)	0.0617 (3)	
N3	0.68920 (14)	0.67197 (11)	0.70931 (11)	0.0599 (3)	
C7	0.58423 (16)	0.73306 (14)	0.73619 (15)	0.0627 (4)	
C8	0.53402 (17)	0.70341 (14)	0.82858 (16)	0.0661 (4)	
C9	0.59378 (16)	0.60379 (13)	0.89864 (14)	0.0559 (4)	
C10	0.70147 (13)	0.53689 (11)	0.87228 (11)	0.0434 (3)	
C11	0.74469 (15)	0.57565 (12)	0.77763 (12)	0.0514 (3)	
C12	0.76845 (14)	0.42525 (11)	0.94349 (11)	0.0462 (3)	
H2A	0.0757 (14)	0.0528 (11)	0.3103 (13)	0.053 (4)*	
H3A	0.2576 (15)	−0.0480 (13)	0.2476 (13)	0.059 (4)*	
H5A	0.1359 (14)	0.1651 (11)	−0.0511 (12)	0.047 (3)*	
H7A	0.5424 (18)	0.8056 (15)	0.6832 (15)	0.076 (5)*	
H8A	0.458 (2)	0.7512 (15)	0.8436 (15)	0.082 (5)*	
H9A	0.5651 (16)	0.5825 (13)	0.9650 (15)	0.069 (5)*	
H11A	0.8211 (16)	0.5323 (13)	0.7567 (12)	0.061 (4)*	
H1N1	−0.0397 (17)	0.2561 (14)	0.0051 (14)	0.069 (4)*	
H1N2	−0.1231 (16)	0.1988 (13)	0.2573 (15)	0.065 (4)*	
H2N2	−0.159 (2)	0.2841 (16)	0.1413 (16)	0.081 (5)*	

### Atomic displacement parameters (Å^2^)

**Table:**

	*U*^11^	*U*^22^	*U*^33^	*U*^12^	*U*^13^	*U*^23^
N1	0.0485 (5)	0.0474 (6)	0.0391 (5)	0.0001 (5)	0.0180 (4)	0.0027 (5)
N2	0.0715 (8)	0.0654 (8)	0.0503 (6)	0.0121 (6)	0.0358 (6)	0.0098 (6)
C1	0.0494 (6)	0.0474 (7)	0.0389 (6)	−0.0066 (5)	0.0191 (5)	−0.0013 (5)
C2	0.0515 (7)	0.0553 (8)	0.0425 (6)	−0.0048 (6)	0.0186 (5)	0.0082 (6)
C3	0.0462 (7)	0.0532 (8)	0.0502 (7)	−0.0018 (6)	0.0150 (6)	0.0085 (6)
C4	0.0437 (6)	0.0549 (8)	0.0461 (7)	−0.0036 (6)	0.0171 (5)	−0.0009 (6)
C5	0.0468 (7)	0.0558 (8)	0.0380 (6)	−0.0039 (6)	0.0183 (5)	0.0002 (6)
C6	0.0611 (8)	0.0880 (11)	0.0618 (9)	0.0186 (8)	0.0268 (7)	0.0076 (8)
O1	0.0818 (7)	0.0743 (7)	0.0557 (6)	0.0160 (5)	0.0417 (5)	0.0135 (5)
O2	0.0788 (6)	0.0671 (6)	0.0499 (5)	0.0249 (5)	0.0358 (5)	0.0115 (4)
N3	0.0668 (7)	0.0543 (7)	0.0576 (7)	0.0011 (6)	0.0199 (6)	0.0071 (6)
C7	0.0590 (8)	0.0479 (8)	0.0726 (10)	−0.0014 (7)	0.0117 (7)	0.0052 (7)
C8	0.0556 (8)	0.0504 (8)	0.0954 (12)	0.0041 (7)	0.0299 (8)	−0.0050 (8)
C9	0.0571 (8)	0.0506 (8)	0.0680 (9)	−0.0019 (6)	0.0318 (7)	−0.0043 (7)
C10	0.0427 (6)	0.0455 (7)	0.0425 (6)	−0.0028 (5)	0.0154 (5)	−0.0048 (5)
C11	0.0545 (7)	0.0530 (8)	0.0495 (7)	0.0018 (6)	0.0214 (6)	0.0005 (6)
C12	0.0519 (7)	0.0514 (8)	0.0376 (6)	0.0001 (6)	0.0185 (5)	−0.0050 (5)

### Geometric parameters (Å, °)

**Table:**

N1—C1	1.3526 (14)	C6—H6B	0.9600
N1—C5	1.3582 (16)	C6—H6C	0.9600
N1—H1N1	0.988 (16)	O1—C12	1.2420 (14)
N2—C1	1.3289 (17)	O2—C12	1.2650 (15)
N2—H1N2	0.883 (16)	N3—C7	1.331 (2)
N2—H2N2	0.936 (19)	N3—C11	1.3354 (17)
C1—C2	1.4073 (18)	C7—C8	1.368 (2)
C2—C3	1.3558 (18)	C7—H7A	1.015 (17)
C2—H2A	0.974 (14)	C8—C9	1.382 (2)
C3—C4	1.4108 (18)	C8—H8A	0.964 (18)
C3—H3A	1.002 (14)	C9—C10	1.3832 (18)
C4—C5	1.3598 (18)	C9—H9A	0.941 (16)
C4—C6	1.4989 (19)	C10—C11	1.3812 (17)
C5—H5A	0.946 (13)	C10—C12	1.5121 (18)
C6—H6A	0.9600	C11—H11A	0.970 (15)
			
C1—N1—C5	122.61 (11)	H6A—C6—H6B	109.5
C1—N1—H1N1	120.1 (8)	C4—C6—H6C	109.5
C5—N1—H1N1	117.3 (8)	H6A—C6—H6C	109.5
C1—N2—H1N2	117.4 (10)	H6B—C6—H6C	109.5
C1—N2—H2N2	119.0 (10)	C7—N3—C11	116.12 (13)
H1N2—N2—H2N2	123.2 (14)	N3—C7—C8	123.94 (14)
N2—C1—N1	118.99 (12)	N3—C7—H7A	115.3 (9)
N2—C1—C2	123.65 (11)	C8—C7—H7A	120.7 (9)
N1—C1—C2	117.35 (11)	C7—C8—C9	118.93 (14)
C3—C2—C1	119.90 (12)	C7—C8—H8A	120.1 (10)
C3—C2—H2A	122.2 (7)	C9—C8—H8A	120.9 (10)
C1—C2—H2A	117.9 (7)	C8—C9—C10	118.82 (14)
C2—C3—C4	121.95 (13)	C8—C9—H9A	121.4 (9)
C2—C3—H3A	120.2 (8)	C10—C9—H9A	119.8 (9)
C4—C3—H3A	117.9 (8)	C11—C10—C9	117.33 (13)
C5—C4—C3	116.37 (12)	C11—C10—C12	121.26 (11)
C5—C4—C6	121.94 (12)	C9—C10—C12	121.41 (12)
C3—C4—C6	121.61 (12)	N3—C11—C10	124.84 (13)
N1—C5—C4	121.81 (12)	N3—C11—H11A	114.9 (8)
N1—C5—H5A	116.7 (8)	C10—C11—H11A	120.2 (8)
C4—C5—H5A	121.5 (8)	O1—C12—O2	124.88 (12)
C4—C6—H6A	109.5	O1—C12—C10	117.64 (11)
C4—C6—H6B	109.5	O2—C12—C10	117.48 (10)
			
C5—N1—C1—N2	179.41 (11)	N3—C7—C8—C9	−0.5 (2)
C5—N1—C1—C2	−0.22 (18)	C7—C8—C9—C10	−0.5 (2)
N2—C1—C2—C3	179.87 (12)	C8—C9—C10—C11	1.1 (2)
N1—C1—C2—C3	−0.52 (18)	C8—C9—C10—C12	−178.48 (12)
C1—C2—C3—C4	0.4 (2)	C7—N3—C11—C10	−0.3 (2)
C2—C3—C4—C5	0.46 (19)	C9—C10—C11—N3	−0.7 (2)
C2—C3—C4—C6	177.42 (13)	C12—C10—C11—N3	178.87 (12)
C1—N1—C5—C4	1.14 (19)	C11—C10—C12—O1	178.34 (12)
C3—C4—C5—N1	−1.21 (18)	C9—C10—C12—O1	−2.15 (19)
C6—C4—C5—N1	−178.16 (12)	C11—C10—C12—O2	−1.73 (19)
C11—N3—C7—C8	0.9 (2)	C9—C10—C12—O2	177.79 (12)

### Hydrogen-bond geometry (Å, °)

**Table:**

*D*—H···*A*	*D*—H	H···*A*	*D*···*A*	*D*—H···*A*
N1—H1N1···O2^i^	0.988 (16)	1.703 (16)	2.6899 (15)	176.8 (16)
N2—H1N2···O2^ii^	0.883 (16)	1.999 (16)	2.8756 (17)	171.7 (15)
N2—H2N2···O1^i^	0.936 (18)	1.878 (18)	2.8122 (17)	176.6 (17)

Symmetry codes: (i) *x*−1, *y*, *z*−1; (ii) *x*−1, −*y*+1/2, *z*−1/2.
